# Commentary: Macrophage IL-1β-positive microvesicles exhibit thrombo-inflammatory properties and are detectable in patients with active juvenile idiopathic arthritis

**DOI:** 10.3389/fimmu.2024.1397527

**Published:** 2024-04-09

**Authors:** Ana T. A. Sachetto, Nigel Mackman

**Affiliations:** Department of Medicine, University of North Carolina at Chapel Hill, Chapel Hill, NC, United States

**Keywords:** microvesicles (MVs), tissue factor (TF), NLRP3-inflammasome, Western blotting, antibody

Rigor and reproducibility in biomedical science are the cornerstones to ensure the quality and reproducibility of preclinical research. Concerns raised about the use of antibodies in common laboratory applications led the International Working Group for Antibody Validation to establish several recommendation for the use of antibodies (1). They suggested that investigators use at least one of five strategies for validation of antibodies for western blotting. The best way to demonstrate that an antibody is correctly recognizing its target is to eliminate or reduce expression of the target protein using genome editing or RNA interference. Unfortunately, most investigators do not independently validate commercial antibodies.

Cambon and colleagues (2) analyzed microvesicles (MVs) in the culture supernatant of THP-1-derived human macrophages and in plasma from patients with systemic juvenile idiopathic arthritis. They showed that macrophages stimulated with bacterial lipopolysaccharide (LPS) and ATP released MVs containing IL-1β and tissue factor (TF). IL-1β-positive MVs were also detected in plasma from patients with systemic juvenile idiopathic arthritis.

The authors used three different methods to measure TF in MVs: electron microscopy, activity and western blotting. The TF activity assay is an established FXa generation assay that uses an inhibitory anti-TF antibody to distinguish between TF-dependent and TF-independent activities (3). The authors observed an increase in MV TF activity in LPS plus ATP-treated macrophages compared with cells treated with LPS alone. In contrast, there was no difference in the levels of TF protein measured by western blotting in LPS-treated cells with or without ATP. We propose that the failure to observe an increase in TF protein using western blotting is due to the antibody used (Abcam, cat. #ab151748). This antibody is a rabbit anti-human monoclonal antibody that was raised against a peptide from human TF. Abcam claimed that it detects human, rat and mouse TF by western blotting. In 2020, we evaluated the ability of different commercial antibodies, including ab151748, to detect human TF by western blotting (4). We found that ab151748 detected an unspecific band in both TF-positive and TF-negative cell lines. Abcam themselves evaluated the ability of ab151748 to detect TF in wild-type HAP1 cells and HAP1 TF knockout cells. They found that it reacted with a similar band in both types of cells and concluded that “ab151748 was not shown to react specifically with the target protein and has therefore been discontinued” (5). We noted that 29 papers (including 15 using western blotting) used this antibody before it was discontinued (4). Since the publication of our paper in 2020, ab151748 have continued to be used for western blotting and other techniques (2, 6–9).

Despite our efforts to alert investigators about the need to independently validate commercial antibodies for the measurement of TF, papers continue to be published with antibodies that do not detect TF.

## Author contributions

AS: Writing – original draft, Writing – review & editing. NM: Writing – review & editing.
